# Clinical, Microbiological and Genomic Characterization of OXA-48-Producing *Klebsiella pneumoniae* ST395 Circulating in a Secondary-Care Hospital in North-Eastern Italy, 2024–2025

**DOI:** 10.3390/antibiotics15090906

**Published:** 2026-09-14

**Authors:** Simone Giuliano, Chiara Moreal, Michela Bulfoni, Jacopo Angelini, Valeria Fox, Nicolò Gualandi, Cinzia Lombardo, Francesco Curcio, Corrado Pipan, Carlo Federico Perno, Francesco Serino, Paolo Cesselli, Carlo Tascini, Paolo Gaibani

**Affiliations:** 1 Infectious Disease Clinic, University Hospital Friuli Centrale, 33100 Udine, Italy; simone.giuliano@asufc.sanita.fvg.it (S.G.); cinzia.lombardo@opbg.net (C.L.); carlo.tascini@uniud.it (C.T.); 2 Department of Medicine, University of Udine, 33100 Udine, Italy; moreal.chiara@spes.uniud.it (C.M.); michela.bulfoni@uniud.it (M.B.); jacopo.angelini@asufc.sanita.fvg.it (J.A.); nicolo.gualandi@uniud.it (N.G.); francesco.curcio@uniud.it (F.C.); corrado.pipan@asufc.sanita.fvg.it (C.P.); 3 Department of Laboratory Medicine, University Hospital Friuli Centrale, 33100 Udine, Italy; 4 Clinical Pharmacology and Toxicology Institute, University Hospital Friuli Centrale ASUFC, 33100 Udine, Italy; 5 Multimodal Laboratory Medicine, Bambino Gesù Children’s Hospital, IRCCS, 00165 Rome, Italy; valeria.fox@opbg.net; 6 Microbiology and Diagnostic Immunology Unit, Bambino Gesù Children’s Hospital, IRCCS, 00165 Rome, Italy; perno@uniroma2.it; 7 UOS Malattie Infettive, U.O.C. Medicina Generale, Portogruaro Hospital, 30026 Venice, Italy; francescosaverio.serino@aulss4.veneto.it; 8 Department of Laboratory Medicine, Azienda Ulss 4 Veneto Orientale, 30026 Portogruaro, Italy; paolo.cesselli@aulss4.veneto.it; 9 Microbiology and Virology Unit, Department of Pathology, Azienda Ospedaliera Universitaria Integrata di Verona, 37134 Verona, Italy; 10 Department of Diagnostic and Public Health, University of Verona, 37134 Verona, Italy

**Keywords:** carbapenemase-producing Enterobacterales, intestinal colonization, sequence type 395, IncL plasmid, genomic surveillance, cefepime/zidebactam

## Abstract

Background/Objectives: This study aimed to characterize the epidemiology, resistance, virulence determinants, and genomic relatedness of OXA-48-producing *Klebsiella pneumoniae* in a secondary-care hospital in north-eastern Italy, and to assess the in vitro activity of cefepime-based β-lactam/β-lactamase inhibitor combinations. Methods: We performed a retrospective observational study of consecutive, non-duplicate isolates (September 2024–January 2025). Susceptibility testing was performed using VITEK^®^ 2 and MIC TestStrip. All isolates underwent single-read Nanopore whole-genome sequencing, with assembly and in silico detection of resistance genes, porin alterations, plasmid replicons and virulence loci. Core-genome single-nucleotide polymorphism analysis with recombination filtering assessed genetic relatedness. Results: Thirteen isolates were recovered from elderly, highly comorbid inpatients, mainly from urine (46.2%) and rectal/fecal screening (38.5%). Twelve patients were classified as colonized, whereas one patient had an infection due to OXA-48-producing *K. pneumoniae*, with the organism recovered from both blood and urine cultures. All belonged to sequence type 395 and carried *bla*OXA-48 and *bla*CTX-M-15, together with a truncated OmpK35 and an OmpK36 loop-3 GD insertion. The yersiniabactin locus ybt16 within integrative conjugative element ICEKp12 was detected in 92.3% of isolates. Core-genome analysis showed high genomic similarity among isolates, consistent with local circulation of an ST395 lineage. Although yersiniabactin may contribute to iron acquisition and bacterial fitness, its presence alone does not establish a hypervirulent phenotype or predict clinical virulence. In the absence of aerobactin, salmochelin, rmpADC, and rmpA2, and without phenotypic virulence testing, these isolates should be regarded as yersiniabactin-positive but non-hypervirulent based on their genomic profile. Core-genome analysis indicated close genetic relatedness among the isolates, consistent with possible local clonal circulation. In a subset, cefepime/enmetazobactam and especially cefepime/zidebactam substantially reduced cefepime minimum inhibitory concentrations. Conclusions: We identified a multidrug-resistant OXA-48-producing *K. pneumoniae* ST395 lineage circulating locally, predominantly in colonization contexts. Active screening and genomic surveillance may support infection control and inform the potential role of emerging cefepime-based inhibitor combinations.

## 1. Introduction

Carbapenemase-producing Enterobacterales (CPE) have emerged as a major global threat, associated with substantial morbidity, mortality, and healthcare costs, particularly among vulnerable patients [1]. Among the various carbapenemases, oxacillinase-48 (OXA-48)-like enzymes have been increasingly reported worldwide and in recent years have emerged in Italy [2]. First described in Turkey, OXA-48-like carbapenemases have subsequently disseminated across European countries and beyond, driven by plasmid transfer and clonal expansion. The epidemiology of OXA-48-producing *K. pneumoniae* remains heterogeneous, driven by both plasmid dissemination and the spread of high-risk clones, while detailed local data integrating genomic, clinical, and therapeutic information remain scarce [3,4]. Multiple OXA-48-like variants (e.g., OXA-181, OXA-232, OXA-244) have been described and are most often carried on highly conserved IncL plasmids, which facilitate interspecies transfer and co-selection with additional resistance determinants [5,6].

In Italy, carbapenem-resistant *Klebsiella pneumoniae* has been mainly associated with the dissemination of KPC-producing high-risk clones; however, recent surveillance indicates an increasing contribution of OXA-48-like producers [7,8,9,10]. Progression from colonization to invasive disease is primarily driven by host factors, but may also vary across enzyme classes and clonal lineages, supporting integrated microbiological, clinical and genomic risk stratification to inform local stewardship [11].

Intestinal colonization with OXA-48-producing *K. pneumoniae* is therefore a key target for surveillance and prevention. Available cohort data suggest that short-term progression to bloodstream infection among OXA-48 carriers is generally low, and that most infections occur early after colonization, supporting risk-stratified approaches rather than universal empiric escalation in all carriers [11]. More recently, in a retrospective cohort of OXA-48-producing Enterobacterales carriers, most infection episodes occurred early after colonization, while low-risk strata identified by clinical scores showed comparatively low 30-day infection rates (<10%), supporting risk-stratified approaches rather than universal empiric escalation in all carriers [12]. In this context, strengthening the integration of genomic findings with clinical and microbiological data is crucial to guide infection prevention strategies and inform the optimal use of novel agents such as cefepime/enmetazobactam in settings where multiple carbapenemase families co-circulate. Recent longitudinal analyses of OXA-48-producing *Klebsiella* have underscored the central role of highly conserved *IncL* (*bla*OXA-48) and *IncFIIK* (*bla*CTX-M) plasmids in sustaining colonization and enabling efficient intra- and interspecies dissemination of *bla*OXA-48/*bla*CTX-M determinants [13]. Based on these premises, this study aimed to characterize OXA-48-producing *K. pneumoniae* isolates belonging to a closely related ST395 lineage detected in a secondary-care hospital in north-eastern Italy by integrating clinical, microbiological, and genomic data to: (i) define the clonal and plasmid background of blaOXA-48, (ii) profile the resistome and virulome, and (iii) describe patient-level features relevant to surveillance and therapeutic decision-making.

## 2. Materials and Methods

### 2.1. Study Cohort

Non-duplicate OXA-48-producing *K. pneumoniae* isolates were recovered during routine diagnostics. Strains included in this study were collected from patients admitted to a secondary care clinical hospital in north-eastern Italy during the period between September 2024 and January 2025. During the study period, all consecutive clinical and surveillance specimens yielding *K. pneumoniae* in routine diagnostics were screened, and OXA-48-producing isolates were identified according to standard laboratory procedures. For the purposes of this analysis, only the first OXA-48-producing *K. pneumoniae* isolate per patient was included; subsequent isolates from the same patient were considered duplicates and excluded.

To determine the clinical significance of isolate recovery, infection was classified according to CDC/NHSN site-specific criteria [14]. Rectal carriage was defined as isolation of OXA-48-producing *K. pneumoniae* from rectal swabs in the absence of signs or symptoms consistent with infection, consistent with previous studies of carbapenem-resistant *K. pneumoniae* rectal carriers [15]. For the purposes of this study, isolates recovered from other non-sterile sites were classified as colonization when the corresponding CDC/NHSN site-specific infection criteria were not fulfilled [14]. For each patient, demographic characteristics, major comorbidities, prior hospitalizations, previous colonization, and exposure to systemic antibiotics in the preceding 12 months were collected. Outcomes at hospital discharge, including in-hospital mortality and, when documented, its attribution to infection, were recorded.

The study protocol was reviewed and approved by the Institutional Review Board of the Department of Medicine, University of Udine (n. 314/2024). The requirement for written informed consent was waived by the Institutional Review Board due to the retrospective design of the study.

### 2.2. Microbiological Characterization

Clinical and surveillance specimens were initially cultured on MacConkey agar. Colonies with morphology suggestive of Enterobacterales were subsequently subcultured on blood agar to obtain pure isolates. Biochemical identification was performed using the VITEK 2 system with the GN identification card (bioMérieux, Marcy-l’Étoile, France). Antimicrobial susceptibility testing was performed using the VITEK 2 AST-N438 and AST-N439 cards, with confirmatory testing using the XN26 card. Carbapenemase production was characterized using the NG-Test CARBA 5 immunochromatographic assay (NG Biotech, Guipry, France). Only the first confirmed OXA-48-producing *Klebsiella pneumoniae* isolate from each patient was included; subsequent isolates from the same patient were considered duplicates and excluded. A multidrug-resistant pathogen was defined as a bacterial isolate resistant to at least one agent in three or more antimicrobial classes.

Results were interpreted following European Committee on Antimicrobial Susceptibility Testing (EUCAST) clinical breakpoints [16]. Susceptibility to cefepime, cefepime/enmetazobactam and cefepime/zidebactam was assessed separately by MIC TestStrip (Liofilchem, Roseto degli Abruzzi, Teramo, Italy) and results were interpreted following the EUCAST and Clinical and Laboratory Standards Institute (CLSI) clinical breakpoints [16,17].

MIC values obtained for cefepime, cefepime/enmetazobactam, and cefepime/zidebactam were compared using a paired Student’s *t*-test, as the three antimicrobial conditions were tested on the same isolates. No correction for multiple comparisons was applied. A two-sided *p*-value < 0.05 was considered statistically significant.

### 2.3. Whole-Genome Sequencing and Resistome Analysis

Whole-genome sequencing of bacterial isolates was performed using the 4bases Microbiome WGS kit (4bases SA, Manno, Switzerland, a shotgun workflow optimized for long-read sequencing on Oxford Nanopore platforms (ONT, Oxford, UK). Libraries were prepared according to the manufacturer’s instructions, using the kit reagents for library construction with Unique Dual Index multiplexing and Nanopore adapters. Sequencing was carried out on GridION instruments using FLO-FLG114 flow cells [18]. Nanopore raw reads were filtered with Filtlong (v0.2.1) to remove reads < 1000 bp and the lowest-quality 10% of reads [19]. Sequencing statistics before and after trimming were evaluated using NanoStat (v1.6.0) [20]. Taxonomic classification was performed with Kraken2 (v2.17.1) to exclude contaminants [21]. De novo genome assembly was conducted with Canu (v2.2) and assembly quality and completeness were assessed with Benchmarking Universal Single-Copy Orthologs (BUSCO) (v6.0.0) using the gammaproteobacteria_odb10 database, and with Quality Assessment Tool for Genome Assemblies (QUAST) (v5.3.0) [22,23,24]. Assembled contigs were annotated using Prokka (v1.14.6, and isolate typing was performed with mlst (v2.25.0) [25,26]. Kleborate (v3.1.0) was used to detect antimicrobial resistance genes, virulence factors, OmpK35/36 mutations, and for K and O typing with Kaptive (v3.0.0b5) [27,28]. Plasmid prediction was performed with MOB-suite (v3.1.8) [29]. Mutations in penicillin-binding proteins (PBPs) were evaluated by mapping reads to pbp1b, pbp1c, pbp2, and pbp3 reference sequences using bwa mem (v0.7.17) [30]. Copy number of the OXA-48 gene was estimated with ccne (v1.1.2) [31].

Nanopore-only sequencing was used because it was the long-read workflow available for this retrospective study and allowed de novo assembly and characterization of resistance, virulence, and plasmid-associated features. Read-quality filtering and assembly-quality assessment were applied to minimize and monitor the potential effects of Nanopore sequencing errors. However, hybrid short- and long-read sequencing was not performed, and this limitation was considered when interpreting fine-scale SNP differences.

### 2.4. Phylogenetic Analysis

The genomic analyses were used for complementary purposes. cgMLST provided a standardized allele-based assessment of relatedness, whereas core-genome SNP analysis was used to evaluate nucleotide-level variation at higher resolution. Bayesian phylogenetic analysis was used as an exploratory assessment of the dated phylogenetic structure of the local isolates and selected publicly available ST395 genomes; it was not intended to establish direct transmission events or definitively determine the geographical origin of the lineage.

Core-genome multilocus sequence typing (cgMLST) was performed using chewBBACA (v3.3.9) with the standard *K. pneumoniae* schema (version 1.0; reference genome NC_012731.1; allelic distance threshold 15) available on the cgMLST platform (https://www.cgmlst.org/ncs/schema/Kpneumoniae2021/, accessed on 1 February 2026). The cgMLST 95 profile (loci present in >95% of strains) was used to generate a pairwise distance matrix, visualized as a Minimum Spanning Tree with Grapetree (v2.2) [32,33].

For comparative phylogenetic analysis, 29 publicly available *K. pneumoniae* ST395 genomes were selected from the NCBI GenBank and European Nucleotide Archive (ENA) databases to provide geographical and temporal context for the local isolates. Eligible genomes were required to have a publicly available accession number, confirmed or in silico-assigned ST395, and available country and year-of-isolation metadata. Accession numbers and associated metadata are reported in Table S1 in the Supplementary Materials. Core-genome SNP alignment was generated with Snippy (v4.6.0) by mapping raw reads from the 13 clinical isolates and the 29 ST395 comparator genomes against the *K. pneumoniae* HS11286 reference genome (GenBank accession CP003200; ST11). HS11286 was used exclusively as the reference genome for SNP calling and was not included among the ST395 comparator genomes. Phylogenetic analysis was conducted using the maximum-likelihood method in IQ-TREE (v2.4.0), applying the best-fit nucleotide substitution model K3Pu+F+ASC+R2 inferred by ModelFinder, with 1000 bootstrap replicates. Pairwise SNP distances were calculated using snp-dists.

Core-genome SNP alignment was generated with Snippy (v4.6.0), mapping raw reads from all 13 clinical isolates and 29 reference genomes of ST395 against the *K. pneumoniae* HS11286 reference strain (RefSeq CP003200) [34]. Phylogenetic analysis was conducted using the Maximum Likelihood (ML) method in IQTREE (v2.4.0), applying the best-fit nucleotide substitution model (K3Pu+F+ASC+R2) inferred by ModelFinder, with 1000 bootstrap replicates. Pairwise SNP distances were calculated with snp-dists [35,36].

As an exploratory assessment of the dated phylogenetic structure, Bayesian phylogenetic inference was performed using BEAST (v1.10.4) based on the core-genome SNP alignment and the available sampling dates [37]. The GTR substitution model inferred by bModelTest was combined with an uncorrelated lognormal relaxed clock and a constant-size coalescent prior [38]. Ten independent BEAST runs were performed, each with 10 million MCMC iterations. Runs were combined, and Tracer (v1.7.1) confirmed an effective sample size (ESS) > 200 for all parameters. Posterior trees were summarized using TreeAnnotator after a 10% burn-in and visualized and annotated using iTOL (v7.4.2) [39,40].

Because a formal temporal-signal assessment was not performed, the Bayesian analysis was considered exploratory and was not used to establish a definitive molecular-clock timescale or geographical origin.

To investigate the transmission chain and infer its putative origin, Bayesian phylogenetic inference was performed using BEAST (v1.10.4) based on the core-genome SNP alignment, incorporating the date of first positivity [37]. The GTR substitution model inferred by bModelTest was combined with an uncorrelated lognormal relaxed clock and a coalescent constant population size prior [38]. Ten independent BEAST runs were performed, each with 10 million MCMC iterations. Runs were combined, and Tracer (v1.7.1) confirmed Effective Sample Size (ESS) > 200 for all parameters [39]. Posterior trees were summarized with TreeAnnotator after 10% burn-in and visualized/annotated with iTOL (v7.4.2) [40].

## 3. Results

### 3.1. Study Cohort and Clinical Characteristics of Patients with OXA-48-Producing K. pneumoniae

Thirteen non-duplicate OXA-48-producing *K. pneumoniae* isolates were recovered between September 2024 and January 2025, each corresponding to a distinct patient (Table 1). Patients were predominantly older adults, with a median age of 81 years (IQR 72–85.5) and an overall age range of 49–89 years; 9/13 (69.2%) were male.

The cohort displayed a high burden of multisystem comorbidity. Chronic cardiovascular disease was present in 8/13 (61.5%) patients, diabetes mellitus in 4/13 (30.8%), and advanced chronic kidney disease in 3/13 (23.1%), including patients receiving long-term hemodialysis. Additional conditions included oncological or hematological disease in 5/13 (38.5%), neurological disorders in 3/13 (23.1%), and chronic respiratory disease in 2/13 (15.4%). A subset of patients had major prior surgery and/or long-term anatomical or device-related risk factors in 3/13 (23.1%).

At the time of the index culture, all thirteen patients were hospitalized but had been admitted from heterogeneous settings: 4/13 (30.8%) were in medical wards, 3/13 (23.1%) in intensive care units, 1/13 (7.7%) in rehabilitation/long-term-care or dialysis facilities, 1/13 (7.7%) in community or protected facilities, and 4/13 (30.8%) were admitted directly from home. Prior healthcare exposure in the preceding 12 months was frequent, with 9/13 (69.2%) patients reporting at least one prior hospital admission. Antimicrobial exposure in the same period was common: 11/13 (84.6%) patients had received systemic antibiotics, often multiple courses of broad-spectrum β-lactams and/or carbapenems.

The isolates were identified mainly through surveillance checks carried out by the organization, rather than specific clinical indications. Detection occurred from urine cultures in 6/13 (46.2%) episodes and from rectal or fecal screening cultures in 5/13 (38.5%); a minority were recovered from blood cultures in 1/13 (7.7%) and from a skin swab at a dialysis catheter exit site in 1/13 (7.7%).

In total, 12 of the 13 patients were classified as colonized with OXA-48-producing *K. pneumoniae*, whereas one patient was classified as having an infection due to the study strain. In this patient, OXA-48-producing *K. pneumoniae* was recovered from both blood and urine cultures during the same clinical episode.

Outcomes reflected the underlying frailty of the cohort, with in-hospital deaths occurring in 5/13 (38.5%), while the remaining patients survived and were discharged.

### 3.2. Phenotypic Characteristics of OXA-48-Producing K. pneumoniae

Overall, the isolate set exhibited a broadly multidrug-resistant phenotype, characterized by high MICs to third- and fourth-generation cephalosporins, carbapenems, fluoroquinolones, and piperacillin/tazobactam, while retaining susceptibility to colistin (Table 2). Specifically, ceftazidime activity was largely lost (12/13 [92.3%] non-susceptible), and both cefotaxime and cefepime were uniformly non-susceptible, with MIC distributions shifted toward the upper end of the testing range. Similarly, all isolates were non-susceptible to meropenem and ciprofloxacin, consistent with a stable resistance backbone across the collection. Amikacin showed only marginal residual activity (susceptible in 1/13 [7.7%]), whereas piperacillin/tazobactam was consistently inactive.

In a subset of isolates (n = 9), addition of enmetazobactam or zidebactam to cefepime resulted in substantial reductions in cefepime MICs compared with cefepime alone across all evaluable strains (see Supplementary Materials in Table S2). In detail, our results demonstrated that in a subset of nine isolates, cefepime/zidebactam showed significantly lower MICs than cefepime alone (*p* < 0.0001) and cefepime/enmetazobactam (*p* < 0.005), based on paired Student’s *t*-tests. No correction for multiple comparisons was applied (Figure S1 in the Supplementary Materials). In particular, cefepime/zidebactam (median 0.38 mg/L, IQR 0.109–0.5 mg/L) exhibited higher antibacterial activity (*p* < 0.0001) than cefepime (median 256 mg/L, IQR 140–256 mg/L) and cefepime/enmetazobactam (median 1 mg/L, IQR 0.625–2 mg/L).

### 3.3. Genomic Characteristics and Local Distribution of OXA-48-Producing Klebsiella pneumoniae

Whole-genome sequencing confirmed that all thirteen isolates belonged to the sequence type (ST) 395, carrying the KL2 capsular locus and O1ab O-antigen locus, consistent with a single, highly homogeneous lineage (Table 3).

Assembly metrics indicated high-quality genomes, with a median genome size of approximately 5.7 Mb, median N50 of ~3.4 × 10^5^ bp and around 5500 predicted genes (Supplementary Materials Table S3). All isolates harbored acquired carbapenemase genes of the blaOXA-48 type; 12/13 (92.3%) carried two copies of blaOXA-48, whereas 1/13 (7.7%) harbored a single copy. In addition, all OXA-48-producing *K. pneumoniae* strains carried *bla*CTX-M-15 and *bla*SHV-182 with the T102S substitution. No mutations in genes coding the main penicillin-binding proteins were detected (*PBP1b*, *PBP1c*, *PBP2* and *PBP3*).

The resistome was highly conserved across the collection, consistent with a stable multidrug-resistance backbone. Most isolates carried aminoglycoside resistance determinants (predominantly *aac(6′)-Ib-cr*, with *aac(3)-Iia* in a subset), together with *sul1*, *tet(A)* and *dfrA1*, supporting broad resistance to aminoglycosides, sulfonamides, tetracyclines and trimethoprim. Plasmid-mediated quinolone resistance was uncommon (*qnrB1* detected in a single isolate), whereas all strains shared the canonical chromosomal substitutions *gyrA* S83I and *parC* S80I, in line with the uniformly high-level fluoroquinolone resistance phenotype. No *mcr* genes or recognized colistin resistance-associated mutations were identified. Outer-membrane permeability alterations were uniform, with a truncated OmpK35 and the characteristic OmpK36 loop-3 Gly134-Asp135 duplication (GD_ins) in all isolates (Table 3).

Virulence profiling showed near-universal carriage of the yersiniabactin locus ybt16 on ICEKp12, whereas the classical hypervirulence determinants aerobactin, salmochelin, rmpADC, and rmpA2 were absent (Supplementary Materials Table S4). The lineage was therefore classified as having a yersiniabactin-positive, non-hypervirulent genomic profile. No specific level of clinical virulence was inferred from these genomic findings alone.

**Figure 1:**
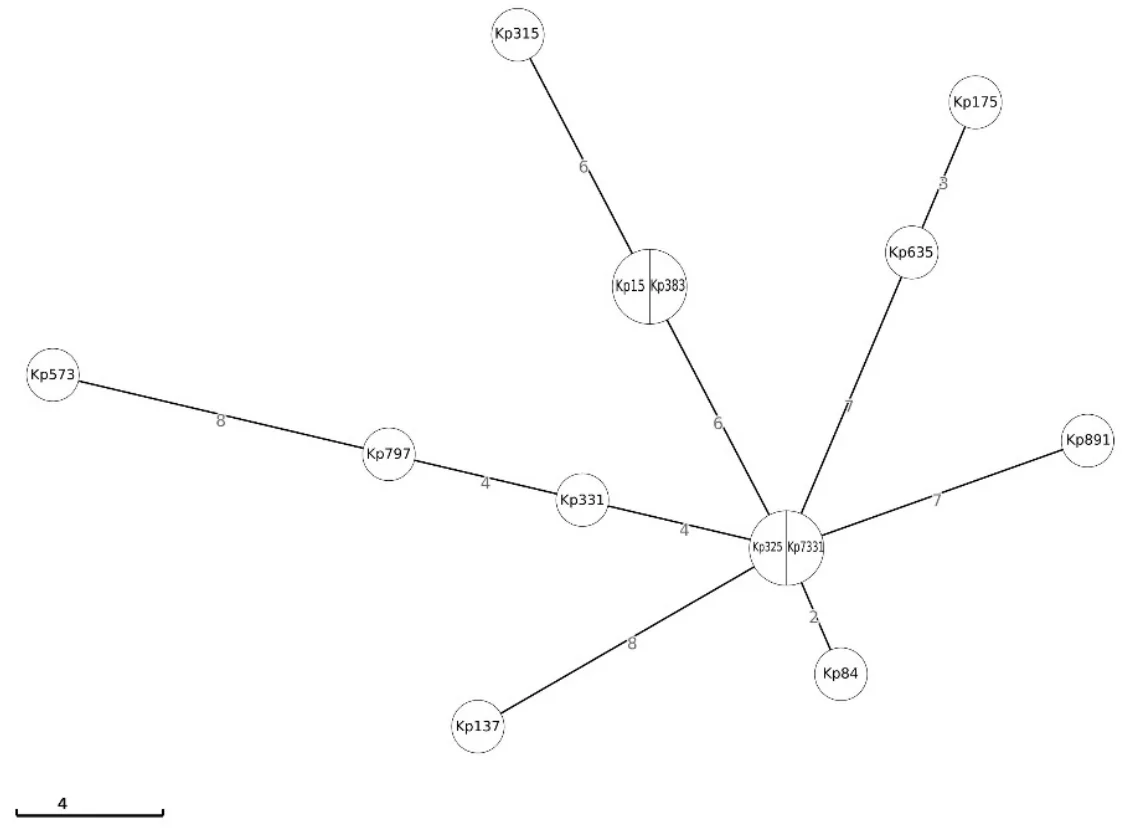
cgMLST-based dendrogram of the OXA-48-producing *K. pneumoniae* isolates included in this study.

Core-genome MLST analysis (2358 loci) revealed close allelic relatedness among isolates, consistent with the local circulation of a genetically related ST395 lineage (Figure 1).

**Figure 2:**
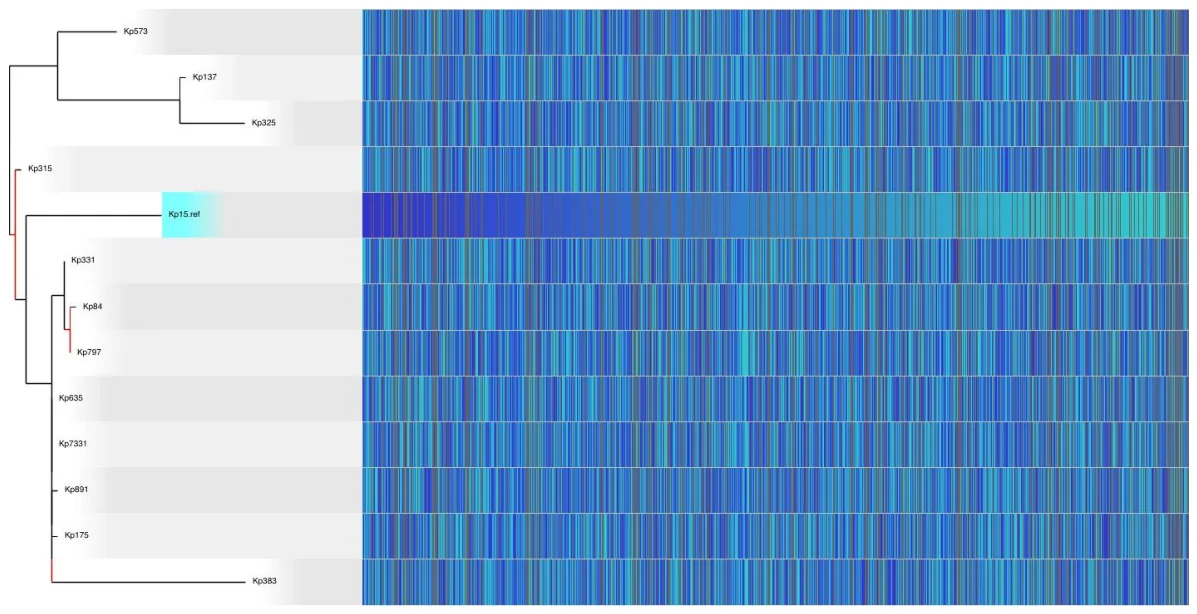
Core-genome SNP-based clustering of OXA-48-producing *K. pneumoniae* isolates. The (**left**) panel shows hierarchical clustering based on the non-recombinant core-genome SNP profile (reference genome included). The (**right**) panel displays the corresponding SNP/variant pattern across the core genome for each isolate (each column represents a variable position/locus), highlighting within-cluster conservation and between-cluster divergence.

The cgMLST dendrogram further indicated limited within-cluster diversification with short branch lengths separating most isolates (Figure S2 in the Supplementary Materials). The minimum spanning tree further resolved fine-scale structure within this cluster: Kp84 occupied a central position, connecting directly to multiple isolates (Kp137, Kp331, Kp573, Kp797, and the split Kp175/635), while other strains (Kp325, Kp891, Kp7331, Kp383, Kp15 and Kp315) branched peripherally with allelic distances ranging from 2 to 27, suggesting minor divergence and potential microevolutionary events. Core-genome SNP analysis of the non-recombinant backbone confirmed these patterns, with a median pairwise distance of 36 SNPs across all isolates. Phylogenetically, OXA-48-producing strains segregated into two clusters: cluster 1, comprising Kp573, Kp137, and Kp325, and cluster 2, comprising nine strains (Figure 2).

**Figure 3:**
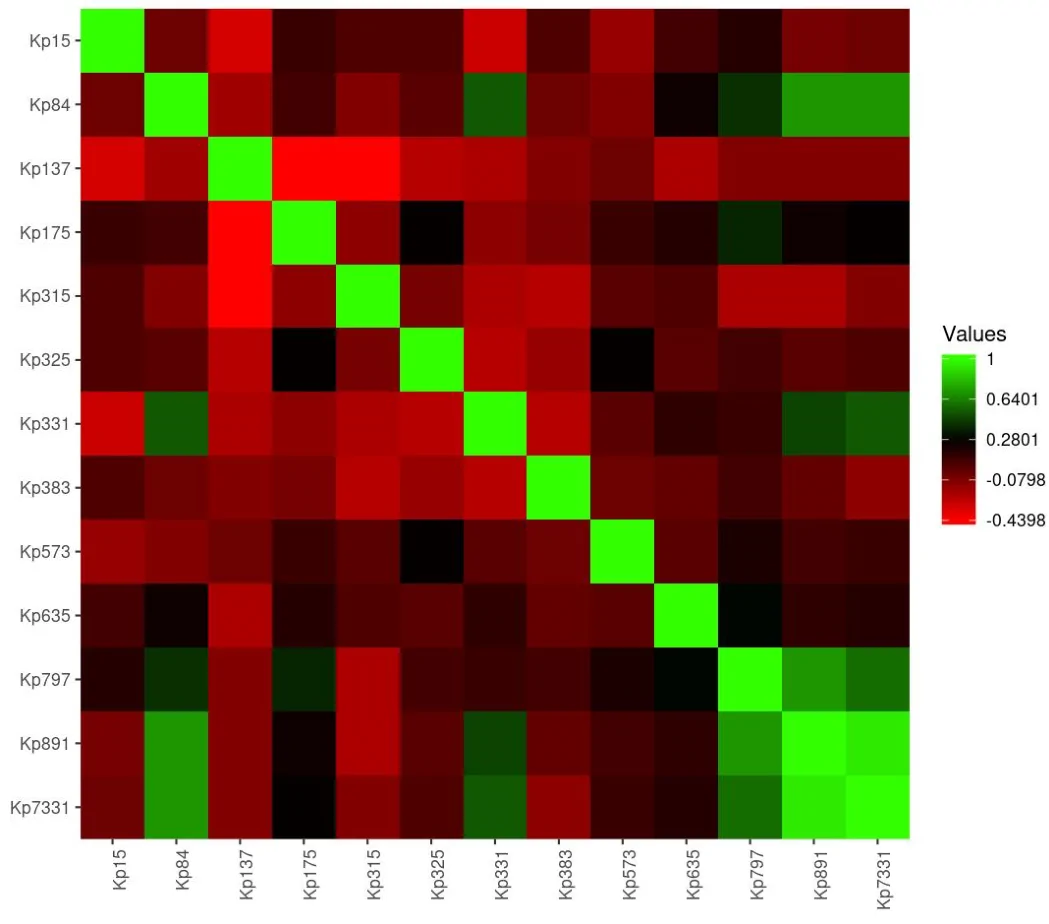
Pairwise similarity matrix among the 13 *K. pneumoniae* isolates, displayed as a heatmap. Rows and columns represent isolates; color intensity reflects the pairwise similarity metric, with 1 indicating self-comparison on the diagonal line.

Median SNP differences were 49 between cluster 1 and the remaining isolates, and 26 within cluster 2, supporting tight clonal relatedness, whereas one isolate (Kp383) displayed greater divergence, differing by 54 SNPs from the core genome of other strains. Together, these analyses highlighted a predominantly clonal lineage with minor sub-clusters and peripheral variants, reflecting potential patterns of genomic relatedness among OXA-48-producing *K. pneumoniae* strains. Correlation analysis based on a pairwise similarity metric further supported higher within-group concordance for selected isolate subsets (e.g., Kp797, Kp891, Kp7331), whereas Kp15 showed comparatively lower similarity to most clinical isolates, consistent with a more divergent genomic profile (Figure 3).

Country and year metadata revealed partial geographic clustering but also evidence of inter-country spread, and recent isolates (2021–2025) tended to carry more AMR genes. Overall, these patterns indicate stable clonal lineages with minor functional variations linked to β-lactam resistance, reflecting the accumulation of resistance and virulence traits across temporally and geographically related isolates.

To assess the phylogenetic topology and estimate divergence times with credibility intervals for each node, a Bayesian approach was applied. The strains were genetically closely related to a ST395 strain isolated in the UK in 2022 (GenBank accession CP133749), which also exhibited the same porin mutations but carried only the SHV gene. In addition, a broader cluster was apparent, encompassing strains isolated in Italy between 2016 and 2021, as well as two strains isolated in Armenia in 2022 (Figure S3 in the Supplementary Materials). The phylogenetic analysis showed genetic relatedness between the local isolates and selected publicly available ST395 genomes from different countries and years. However, given the limited dataset and the available epidemiological information, these findings cannot determine the geographical origin of the lineage or establish the direction or specific events of transmission.

## 4. Discussion

This study identified the local circulation of genetically related OXA-48-producing *K. pneumoniae* ST395 isolates at a hospital in north-eastern Italy, predominantly among elderly, highly comorbid patients with extensive previous healthcare exposure. All isolates belonged to the ST395 KL2/O1ab lineage and had similar resistome profiles. The study provided a genomic overview of OXA-48-producing *K. pneumoniae* in a setting historically dominated by KPC-producing clones, contributing to the still limited Italian evidence on OXA-48 epidemiology [3,7,8]. The identification of a highly homogeneous ST395 lineage is consistent with reports of ST395 as a high-risk clone associated with OXA-48 and CTX-M-15 in Europe and beyond [6]. Previous studies reported similar epidemiological spread, describing the emergence of OXA-48-producing ST395 and clonal diffusion of this sequence type in the region [41]. Genomic correlation analysis revealed that all strains included in this study were clonally related. Core-genome SNP analysis identified two closely related subclusters, with one isolate (Kp383) segregating as a more divergent branch while remaining within the same clade. These findings are consistent with the local circulation of a closely related ST395 OXA-48 lineage within the healthcare setting. However, the source and timing of its introduction, as well as direct transmission events between patients, cannot be determined from the available epidemiological and genomic data. Accordingly, the possibility that these isolates represent sporadic detections of a closely related lineage already circulating locally, rather than documented patient-to-patient transmission, cannot be excluded. Previous epidemiological studies showed that OXA-48-producing ST395 *K. pneumoniae* has emerged recently in different European countries, reinforcing the notion that this clone has become an established component of the regional carbapenem-resistant Enterobacterales landscape [42,43].

This clinical profile, characterized by multimorbidity, prolonged healthcare exposure and repeated antibiotic pressure, aligns with host risk factors most frequently associated with the acquisition and persistence of carbapenemase-producing Enterobacterales in European surveillance and epidemiological investigations [1,10]. In the present series, most OXA-48-producing *K. pneumoniae* isolates were detected in the context of colonization rather than clinically documented infection, with isolates mainly recovered from urine or rectal/fecal screening. However, one patient was classified as having an infection due to the study strain, with OXA-48-producing *K. pneumoniae* recovered from both blood and urine cultures during the same clinical episode. This predominance of colonization over infection is consistent with published evidence indicating that carriage of OXA-48-producing Enterobacterales does not inevitably lead to infection and that progression may be uncommon, particularly outside high-risk groups [11]. Notably, this contrasts with infection-focused cohorts in which OXA-48 production has been associated with a higher proportion of urinary-tract infection episodes compared with non-OXA-48 *K. pneumoniae* (35.0% vs. 14.5%; *p* < 0.0001) [44]. This apparent discrepancy likely reflects, at least in part, differences in clinical case ascertainment and culture practices in frail, device-exposed inpatients, in whom non-specific deterioration and frequent sampling, especially from the urinary tract, can increase the likelihood of detecting carriage without a compatible infectious syndrome. Consequently, microbiological positivity at non-sterile sites should not be equated with infection in the absence of syndrome-based criteria, and careful clinical adjudication remains essential to avoid overestimating infection burden among OXA-48 carriers [11,44].

Consequently, the management of colonized patients who develop suspected infection remains controversial, as clinicians must balance the risk of inadequate empirical therapy against the harm of unnecessary broad-spectrum or novel agents. Recent work specifically focusing on OXA-48 carriers supports the use of clinical risk scores to identify patients at low risk in whom routine empirical escalation may be avoidable, while recognizing that infection risk is concentrated in high-risk profiles and early after colonization [12]. However, commonly used prognostic scores were originally developed in KPC-dominated and carbapenemase-producing Enterobacterales bloodstream infection cohorts and before the widespread availability of newer β-lactams, which may limit their direct applicability [15,45,46]. In this study, only one patient had a clinically documented OXA-48-producing *K. pneumoniae* infection, and no prior colonization with the study strain was documented in this case. Therefore, progression from colonization to infection could not be evaluated. This finding reinforces the importance of diagnostic stewardship and clear case definitions when interpreting positive cultures in high-risk carriers.

Crude in-hospital mortality occurred in a highly frail population with a substantial burden of comorbidities and competing acute illnesses. Given the single documented OXA-48-producing *K. pneumoniae* infection and the lack of evidence supporting organism-attributable mortality, no conclusions can be drawn regarding mortality attributable to the study strain. Against this background, the in vitro activity of cefepime-based combinations in the outbreak cluster of genetically related isolates was explored.

In vitro testing of cefepime/enmetazobactam and cefepime/zidebactam against OXA-48-producing isolates showed substantial reductions in cefepime MICs compared with cefepime alone, consistent with inhibitor/enhancer-driven potentiation of the cefepime backbone; cefepime/zidebactam generally achieved lower MICs than cefepime/enmetazobactam. However, the observed MIC reductions should not be interpreted as evidence of pharmacokinetic/pharmacodynamic target attainment or clinical efficacy. Further PK/PD and clinical studies are required to assess the clinical relevance of these findings. This observation is biologically plausible given that OXA-48-like enzymes exhibit limited hydrolytic activity against expanded-spectrum cephalosporins, including ceftazidime and, to an even greater extent, cefepime, and therefore do not constitute the primary determinant of resistance to these agents [47,48,49]. Instead, reduced susceptibility to expanded-spectrum cephalosporins in OXA-48-producing isolates is largely driven by ESBL co-production, most commonly CTX-M-type enzymes, often in combination with outer-membrane permeability defects. In our collection, the co-occurrence of *bla*CTX-M-15 and porin alterations is consistent with the high cephalosporin MICs observed [49]. Accordingly, the observed activity of cefepime-based combinations is primarily determined by the partner compound, which counteracts co-produced ESBLs and other serine β-lactamases, thereby enabling cefepime to retain activity against OXA-48-producing strains. Our findings are consistent with non-clinical data showing potent activity of cefepime/enmetazobactam against ESBL- and AmpC-producing *Enterobacterales* and subsets of CRE, including some OXA-48 producers, as well as with the phase 3 ALLIUM trial, in which cefepime/enmetazobactam was superior to piperacillin/tazobactam for complicated urinary tract infections and acute pyelonephritis [18,50,51]. The retained in vitro activity of both cefepime/enmetazobactam and cefepime/zidebactam in this context supports their potential for further evaluation as carbapenem-sparing options for at least some OXA-48-producing *K. pneumoniae* lineages.

Nonetheless, several limitations require caution. The number of isolates tested was small, and the study did not correlate the MICs of these agents with clinical outcomes during cefepime/enmetazobactam therapy. Given the small number of isolates tested with the cefepime-based combinations (n = 9), these findings should be considered exploratory and hypothesis-generating. The observed MIC reductions indicate in vitro activity but cannot by themselves support conclusions regarding clinical efficacy or optimal dosing.

A further limitation is that sequencing was performed exclusively using Oxford Nanopore long reads. Although quality filtering and assembly-quality assessment were applied, the higher per-read error rate of Nanopore sequencing compared with Illumina or hybrid sequencing may affect fine-scale SNP resolution. Therefore, the observed genomic relatedness should be interpreted as supporting possible local circulation rather than demonstrating direct patient-to-patient transmission. Moreover, the small number of local isolates and the restricted sampling period constrain the reliability of molecular-clock inference. Formal clinical breakpoints for cefepime/enmetazobactam and cefepime/zidebactam against OXA-48-producing *K. pneumoniae* are not yet fully established, and current interpretation relies on PK/PD considerations and extrapolation from other settings. In addition to standard contact precautions and rectal screening, serial microbiological and, where feasible, genomic monitoring of high-risk carriers receiving ceftazidime/avibactam or other novel BL/BLI combinations may help detect early resistance evolution, although this warrants prospective evaluation and may help prevent onward transmission of more resistant sublineages. Because detailed patient-level epidemiological information, including ward overlap, healthcare pathways, admission and discharge timelines, and direct contact tracing, was not systematically available, the study cannot establish specific transmission events or definitively distinguish an outbreak from the sporadic detection of closely related strains circulating within the local healthcare setting. In this context, our data should be considered exploratory, hypothesis-generating evidence supporting further clinical evaluation of cefepime/enmetazobactam and cefepime/zidebactam against OXA-48 producers, rather than evidence to reposition these agents as standard of care.

## 5. Conclusions

In summary, this study documented the local circulation of a multidrug-resistant OXA-48-producing *K. pneumoniae* ST395 KL2/O1ab lineage in a secondary-care hospital in north-eastern Italy. The isolates shared a highly similar genomic background characterized by *bla*OXA-48, *bla*CTX-M-15, *bla*OXA-1 and conserved porin alterations. Most detections occurred in colonization contexts, although one patient had a documented infection due to the study strain. Consistent with the frequent co-occurrence of ESBL determinants and porin defects, cefepime MICs were high when tested alone. However, in vitro testing in a subset of isolates showed marked MIC reductions with cefepime/enmetazobactam and cefepime/zidebactam compared with cefepime alone; notably, the magnitude of MIC lowering was not identical across the two combinations, with cefepime/zidebactam generally achieving lower MICs than cefepime/enmetazobactam. At the same time, the preserved in vitro activity of cefepime/enmetazobactam and cefepime/zidebactam supports further clinical evaluation of these combinations as potential carbapenem-sparing options in settings where OXA-48 is circulating. The identification of a high-risk ST395 lineage among these genetically related isolates further underscores the importance of locally tailored antimicrobial stewardship programmes integrated with infection-control measures to reduce continued circulation and prevent the establishment of long-term reservoirs within healthcare settings. However, these findings should be regarded as exploratory and hypothesis-generating given the small sample size and the absence of established clinical breakpoints and outcome data.

## Figures and Tables

**Table 1:** Clinical features of patients with OXA-48-producing *Klebsiella pneumoniae* strains.

Isolate	Material	Gender, Age (Years)	Comorbidities	Admission	Previous Colonization	Infection/Colonization by OXA-48-Kp	Antibiotic Therapy	Outcome
15	Rectal swab	M, 84	Diabetes Chronic kidney failure	ICU	*Proteus mirabilis*	Colonization	Piperacillin/Tazobactam	Dead
84	Rectal swab	M, 87	Epilepsy Cerebral amyloid angiopathy Intracranial hemorrhage	Protected facilities	-	Colonization	Amoxicillin/clavulanic acid Ceftiaxon Piperacillin/Tazobactam	Survived
137	Rectal swab	F, 75	COPD Diverticulosis	Home	-	Colonization	Ceftriaxon Meropenem	Survived
175	Urine	M, 86	Chronic ischemic cardiomyopathy Waldenström’s macroglobulinemia Anemia Diabetes	Home	-	Colonization	-	Dead
315	Blood culture *	F, 81	Diabetes Chronic ischemic cardiomyopathy Atrial fibrillation	Medical ward	*Escherichia coli*	Infection	Amoxicillin/clavulanic acid Ceftiaxon Piperacillin/Tazobactam	Dead
325	Urine	F, 85	Hypertension Monoclonal gammopathy of uncertain significance	Home	-	Colonization	Ceftriaxon	Survived
331	Urine	F, 72	Hypertension Diverticulosis	ICU	MSSA	Colonization	Ceftriaxon Daptomycin Oxacillin	Dead
383	Rectal swab	M, 84	Hypertension Chronic kidney failure	Medical ward	-	Colonization	Amoxicillin/clavulanic acid	Survived
573	Urine	M, 72	Diabetes Prostate carcinoma	Medical ward	-	Colonization	Cefazolin Piperacillin/Tazobactam	Survived
635	Urine	M, 79	Hypertension Atrial fibrillation	Medical ward	MRSA	Colonization	Teicoplanin	Survived
797	Skin swab	M, 49	Hypertension Chronic kidney failure Antiphospholipid syndrome	Medical ward (dialysis)	MRSA	Colonization	Clindamycin	Survived
891	Urine	M, 89	Hypertension Atrial fibrillation Dementia	Home	-	Colonization	-	Survived
7331	Rectal swab	M, 65	Glioblastoma Asthma	ICU	-	Colonization	Ceftriaxon Piperacillin/Tazobactam Vancomycin	Death

* OXA-48-producing *K. pneumoniae* was also recovered from urine during the same clinical episode. Legend: COPD, chronic obstructive pulmonary disease; F, female; ICU, intensive care unit; M, male; MSSA, methicillin-sensitive *Staphylococcus aureus*; MRSA, methicillin-resistant *Staphylococcus aureus*.

**Table 2:** In vitro activity of antimicrobials against OXA-48-producing *Klebsiella pneumoniae* isolates included in this study.

Antimicrobial Agent	MICs Range mg/L	MIC50 (mg/L)	MIC90 (mg/L)	EUCAST C.B. Interpretation ^i^		
				Susceptible (%)	Susceptible Increased Exposure (%)	Resistant (%)
AK	≤1–8	4	8	1/13 (7.7)	NA	12/13 (92.3)
CAZ	2–≥64	≥64	≥64	0/13 (0)	1/13 (7.7)	12/13 (92.3)
CIP	≥4	≥4	≥4	0/13 (0)	0/13 (0)	13/13 (100)
COL	0.5	0.5	0.5	13/13 (100)	NA	0/13 (0)
CTX	≥16–≥64	≥64	≥64	0/13 (0)	0/13 (0)	13/13 (100)
FEP	8–≥32	≥32	≥32	0/13 (0)	0/13 (0)	13/13 (100)
MEM	≥16	≥16	≥16	0/13 (0)	0/13 (0)	13/13 (100)
PIP/TAZ	≥128	≥128	≥128	0/13 (0)	NA	13/13 (100)
SXT	≥320	≥320	≥320	0/13 (0)	NA	13/13 (100)

^i^ MICs interpreted using EUCAST Clinical Breakpoint tables for interpretation of MICs and zone diameters. Version 16.0, 2026. https://www.eucast.org (accessed on 1 February 2026). Abbreviations: AK, Amikacin; CAZ, Ceftazidime; CIP, Ciprofloxacin; COL, Colistin; CTX, Cefotaxime; EUCAST, European Committee on Antimicrobial Susceptibility Testing; FEP, Cefepime; MEM, Meropenem; NA, Not applicable; PIP/TAZ, Piperacillin/Tazobactam; SXT, Trimethoprim/Sulfamethoxazole.

**Table 3:** Resistome characteristics of OXA-48-producing *Klebsiella pneumoniae* included in this study.

Isolate	Material	Serotype		PBP	Porins		Antimicrobial Resistance Genes											
		O-Antigen Locus	Capsular Locus		OmpK35 ^i^	OmpK36GD ^ii^	Aminoglycosides	β-Lactamases	Colistin	Fluoroquinolones	Fosfomycin	Glycopeptide	Phenicol	Rifampicin	Sulfonamide	Tetracycline	Tigecycline	Trimethoprim
15	Rectal swab	O1ab	KL2	−	truncated (aa260; 72%)	+	*aac(6′)-Ib-cr*	*bla*SHV-182 T102S; *bla*OXA-1; *bla*CTX-M-15; *bla*OXA-48 (two copies)	−	*gyrA* S83I; *parC* S80I	−	−	*catB4*; *catA1*	−	*sul1*	*tet(A)*	−	*dfrA1*
84	Rectal swab	O1ab	KL2	−	truncated (aa260; 72%)	+	*aac(6′)-Ib-cr*	*bla*SHV-182 T102S; *bla*OXA-1; *bla*CTX-M-15; *bla*OXA-48 (two copies)	−	*gyrA* S83I; *parC* S80I	−	−	*catB4*; *catA1*	−	*sul1*	*tet(A)*	−	*dfrA1*
137	Rectal swab	O1ab	KL2	−	truncated (aa260; 72%)	+	*aac(3)-Iia*; *aac(6′)-Ib-cr*	*bla*SHV-182 T102S; *bla*OXA-1; *bla*CTX-M-15; *bla*OXA-48 (two copies)	−	*gyrA* S83I; *parC* S80I (with mutation qnrB1)	−	−	*catB4*; *catA1*	−	*sul1*	*tet(A)*	−	*dfrA1*
175	Urine	O1ab	KL2	−	truncated (aa260; 72%)	+	*aac(3)-Iia*; *aac(6′)-Ib-cr*	*bla*SHV-182 T102S; *bla*OXA-1; *bla*CTX-M-15; *bla*OXA-48 (two copies)	−	*gyrA* S83I; *parC* S80I	−	−	*catB4*; *catA1*	−	*sul1*	*tet(A)*	−	*dfrA1*
315	Blood culture	O1ab	KL2	−	truncated (aa260; 72%)	+	*aac(6′)-Ib-cr*	*bla*SHV-182 T102S; *bla*OXA-1; *bla*CTX-M-15; *bla*OXA-48 (two copies)	−	*gyrA* S83I; *parC* S80I	−	−	*catB4*; *catA1*	−	*sul1*	*tet(A)*	−	*dfrA1*
325	Urine	O1ab	KL2	−	truncated (aa260; 72%)	+	*aac(3)-Iia*; *aac(6′)-Ib-cr*	*bla*SHV-182 T102S; *bla*OXA-1; *bla*CTX-M-15; *bla*OXA-48 (two copies)	−	*gyrA* S83I; *parC* S80I	−	−	*catB4*; *catA1*	−	*sul1*	*tet(A)*	−	*dfrA1*
331	Urine	O1ab	KL2	−	truncated (aa260; 72%)	+	*aac(6′)-Ib-cr*	*bla*SHV-182 T102S; *bla*OXA-1; *bla*CTX-M-15; *bla*OXA-48 (two copies)	−	*gyrA* S83I; *parC* S80I	−	−	*catB4*; *catA1*	−	*sul1*	*tet(A)*	−	*dfrA1*
383	Rectal swab	O1ab	KL2	−	truncated (aa260; 72%)	+	*aac(6′)-Ib-cr*	*bla*SHV-182 T102S; *bla*OXA-1; *bla*CTX-M-15; *bla*OXA-48 (two copies)	−	*gyrA* S83I; *parC* S80I	−	−	*catB4*; *catA1*	−	*sul1*	*tet(A)*	−	*dfrA1*
573	Urine	O1ab	KL2	−	truncated (aa260; 72%)	+	*aac(3)-Iia*; *aac(6′)-Ib-cr*	*bla*SHV-182 T102S; *bla*OXA-1; *bla*CTX-M-15; *bla*OXA-48 (two copies)	−	*gyrA* S83I; *parC* S80I	−	−	*catB4*	−	*sul1*	*tet(A)*	−	*dfrA1*
635	Urine	O1ab	KL2	−	truncated (aa260; 72%)	+	*aac(3)-Iia*; *aac(6′)-Ib-cr*	*bla*SHV-182 T102S; *bla*OXA-1; *bla*CTX-M-15; *bla*OXA-48 (two copies)	−	*gyrA* S83I; *parC* S80I	−	−	*catB4*; *catA1*	−	*sul1*	*tet(A)*	−	*dfrA1*
797	Skin swab	O1ab	KL2	−	truncated (aa260; 72%)	+	−	*bla*SHV-182 T102S; *bla*OXA-1; *bla*CTX-M-15; *bla*OXA-48	-	*gyrA* S83I; *parC* S80I	−	−	−	−	−	−	−	−
891	Urine	O1ab	KL2	−	truncated (aa260; 72%)	+	−	*bla*SHV-182 T102S; *bla*OXA-1; *bla*CTX-M-15; *bla*OXA-48 (two copies)	-	*gyrA* S83I; *parC* S80I	−	−	*catA1*	−	*sul1*	*tet(A)*	−	*dfrA1*
7331	Rectal swab	O1ab	KL2	−	truncated (aa260; 72%)	+	*aac(3)-Iia*; *aac(6′)-Ib-cr*	*bla*SHV-182 T102S; *bla*OXA-1; *bla*CTX-M-15; *bla*OXA-48 (two copies)	−	*gyrA* S83I; *parC* S80I	−	−	*catB4*; *catA1*	−	*sul1*	*tet(A)*	−	*dfrA1*

^i^ 72% indicates predicted protein length relative to the expected full-length OmpK35 reference. ^ii^ OmpK36GD indicates Gly134-Asp135 duplication in loop 3. Abbreviations: +, Present; −, Absent; PBP, Penicillin-binding protein.

## Data Availability

The clinical and genomic data supporting this report are contained within the article and Supplementary Materials. Raw sequencing reads are available upon reasonable request.

## Supplementary Materials

The following supporting information can be downloaded at https://www.mdpi.com/article/10.3390/antibiotics15090906/s1, References [8,52,53] are cited in the Supplementary Materials.
