# Supplementary material for: Clinical, Microbiological and Genomic Characterization of OXA-48-Producing Klebsiella pneumoniae ST395 Circulating in a Secondary-Care Hospital in North-Eastern Italy, 2024–2025
Source: Antibiotics (Basel). 2026 Sep 14;15(9):906. doi: 10.3390/antibiotics15090906 (PMC13603657; doi:10.3390/antibiotics15090906)
Supplement: Supplementary file 1 [file antibiotics-15-00906-s001.zip › antibiotics-4566712-supplementary.pdf]

**Table S1. Publicly available *Klebsiella pneumoniae* ST395 genomes included in the comparative phylogenetic analysis.** The table reports accession number, strain name, country, year of isolation, specimen source, sequence type, capsular locus, O-antigen locus, porin alterations, and detected resistance and virulence determinants. *K. pneumoniae* HS11286 (CP003200; ST11), used exclusively as the reference genome for SNP calling, is reported separately and was not counted among the 29 ST395 comparator genomes.

| GenBank/ ENA accession | Strain | Country/location | Year | Specimen source | ST | K locus | O locus | OmpK alterations | Detected β-lactamases | Yersiniabactin | Aerobactin (iuc) | rmpADC | rmpA2 | Reference |
|---|---|---|---|---|---|---|---|---|---|---|---|---|---|---|
| GCA_902703855 | Kpngiani7132576 | Catania, Italy | 2016 | blood | 395 | KL2 | O1ab | OmpK35-72% (truncation at aa 260) | TEM-1D; OXA-1; CTX-M-15; KPC-3; SHV-11 | ybt 16; ICEKp12 | iuc 1 | Not detected | rmpA2_6*-47% | [8] |
| GCA_902703865 | Kpngiani7132575 | Catania, Italy | 2016 | blood | 395 | KL2 | O1ab | OmpK35-72% (truncation at aa 260) | TEM-1D; OXA-1; CTX-M-15; OXA-48; SHV-11 | ybt 16; ICEKp12 | iuc 1 | Not detected | Not detected | [8] |
| GCA_902703905 | Kpngiani7132580 | Catania, Italy | 2016 | blood | 395 | KL2 | O1ab | OmpK35-72% (truncation at aa 260) | TEM-1D; OXA-1; CTX-M-15; KPC-3; SHV-11 | ybt 16; ICEKp12 | iuc 1 | Not detected | rmpA2_6*-47% | [8] |
| GCA_902703965 | Kpngiani7132573 | Catania, Italy | 2016 | blood | 395 | KL2 | O1ab | OmpK35-72% (truncation at aa 260) | TEM-1D; OXA-1; CTX-M-15; KPC-3; SHV-11 | ybt 16; ICEKp12 | iuc 1 | Not detected | rmpA2_6*-47% | [8] |
| GCA_902703995 | Kpngiani7132578 | Catania, Italy | 2016 | blood | 395 | KL2 | O1ab | OmpK35-72% (truncation at aa 260) | TEM-1D; OXA-1; CTX-M-15; KPC-3; SHV-11 | ybt 16; ICEKp12 | iuc 1 | Not detected | Not detected | [8] |
| GCA_902704025 | Kpngiani7132574 | Catania, Italy | 2016 | blood | 395 | KL2 | O1ab | OmpK35-72% (truncation at aa 260) | TEM-1D; OXA-1; CTX-M-15; KPC-3; SHV-11 | ybt 16; ICEKp12 | iuc 1 | Not detected | rmpA2_6*-47% | [8] |
| GCA_902704055 | Kpngiani7132594 | Firenze, Italy | 2016 | blood | 395 | KL2 | O1ab | OmpK35-72% | TEM-1D; | ybt 16; ICEKp12 | iuc 1 | Not detected | rmpA2_6*-47% | [8] |

| | | | | | | | | | | | | | | |
|---|---|---|---|---|---|---|---|---|---|---|---|---|---|---|
| | | | | | | | | (truncation at aa 260) | KPC-3; SHV-11 | | | | | |
| GCA_902704255 | Kpngiani7132621 | Torino, Italy | 2016 | blood | 395 | KL2 | O1ab | OmpK35-72% (truncation at aa 260) | OXA-48; SHV-11 | ybt 16; ICEKp12 | iuc 1 | Not detected | rmpA2_6*-47% | [8] |
| CP009114.1 | BlaNDM-1 | USA | 2012 | Urine | 395 | KL108 | O1ab | OmpK35-72% (truncation at aa 260) | SHV-11 | Not detected | Not detected | Not detected | Not detected | Not reported |
| CP018364.1 | Kp_Goe_62629 | Germany | 2015 | Urine | 395 | KL108 | O1ab | OmpK35-72% (truncation at aa 260) | SHV-11 | Not detected | Not detected | Not detected | Not detected | Not reported |
| CP061961.1 | JNK002 | China | 2015 | Urine | 395 | KL39 | O1ab | OmpK35-72% (truncation at aa 260);OmpK36GD | SHV-11 | ybt 16; ICEKp12 | Not detected | Not detected | Not detected | Not reported |
| CP071086.1 | 3347689I | Switzerland | 2020 | outer malleolus | 395 | KL108 | O1ab | OmpK35-72% (truncation at aa 260) | TEM-1D; NDM-1; SHV-11 | ybt 15; ICEKp11 (truncated) | Not detected | Not detected | Not detected | Not reported |
| CP082240.1 | GIMC1002:Kpn-3Ts64 | Russia | 2016 | sputum | 395 | KL39 | O1ab | OmpK35-72% (truncation at aa 260) | SHV-11 | ybt 16; ICEKp12 | Not detected | Not detected | Not detected | Not reported |
| CP120890.1 | GIMC1006:Kpn-102106 | Russia | 2021 | blood | 395 | KL2 | O1ab | OmpK35-72% (truncation at aa 260);OmpK36GD | SHV-11 | ybt 14; ICEKp12 | Not detected | Not detected | Not detected | Not reported |
| CP122342.1 | SCCH112:Kpn2161521 | Russia | 2021 | anus swab | 395 | KL39 | O1ab | OmpK35-72% (truncation at aa 260) | SHV-11 | ybt 16; ICEKp12 | Not detected | Not detected | Not detected | Not reported |
| CP122350.1 | SCCH87:Kpn2182174 | Russia | 2021 | blood | 395 | KL2 | O1ab | OmpK35-72% (truncation at aa 260) | SHV-11 | ybt 16; ICEKp12 | Not detected | Not detected | Not detected | Not reported |
| CP122356.1 | SCCH114:Kpn2161584 | Russia | 2021 | sputum | 395 | KL39 | O1ab | OmpK35-72% (truncation at aa 260) | SHV-11 | ybt 16; ICEKp12 | Not detected | Not detected | Not detected | Not reported |

| | | | | | | | | | | | | | | |
|---|---|---|---|---|---|---|---|---|---|---|---|---|---|---|
| CP127511.1 | ER29693.4 | USA | 2014 | blood | 395 | KL64 | O1ab | OmpK35-32% (truncation at aa 115) | SHV-11 | ybt 16; ICEKp12 | Not detected | Not detected | Not detected | Not reported |
| CP132631 | 110821 | Germany | 2022 | surveillance swab | 395 | KL39 | O1ab | OmpK35-72% (truncation at aa 260);OmpK36GD | SHV-11 | ybt 16; ICEKp12 | Not detected | Not detected | Not detected | Not reported |
| CP133749.1 | KP17 | UK | 2022 | rectal swab | 395 | KL2 | O1ab | OmpK35-72% (truncation at aa 260);OmpK36GD | SHV-11 | Not detected | Not detected | Not detected | Not detected | Not reported |
| CP135508.1 | isolateK | Canada | 2020 | Not reported | 395 | KL64 | O1ab | OmpK35-32% (truncation at aa 115) | SHV-11 | ybt 16; ICEKp12 | Not detected | Not detected | Not detected | Not reported |
| CP168936.1 | KpA481 | Armenia | 2022 | Urine | 395 | KL2 | O2a | OmpK35-72% (truncation at aa 260) | TEM-1D; OXA-1; CTX-M-15; NDM-1; SHV-11 | ybt 16; ICEKp12 | iuc 1 | rmp 1; KpVP-1 | rmpA2_6*-47% | Not reported |
| CP168937.1 | KpA285 | Armenia | 2022 | Urine | 395 | KL2 | O2a | OmpK35-72% (truncation at aa 260) | TEM-1D; OXA-1; CTX-M-15; NDM-1; SHV-11 | ybt 16; ICEKp12 | iuc 1 | Not detected | rmpA2_6*-47% | Not reported |
| ERR1217208 | 18174_5 | Le Grazie, Italy | 2013 | Wound Discharge | 395 | KL108 | O1ab | OmpK35-72% (truncation at aa 260) | TEM-1D; OXA-1; CTX-M-15; SHV-11 | Not detected | Not detected | Not detected | Not detected | Not reported |
| GCA_000821985.1 | 39AVR | Milan, Italy | 2014 | Not reported | 395 | KL108 | O1ab | OmpK35-72% (truncation at aa 260) | CTX-M-15; SHV-11 | Not detected | Not detected | Not detected | Not detected | [52] |
| GCA_000822125.1 | 46AVR | Milan, Italy | 2014 | Not reported | 395 | KL108 | O1ab | OmpK35-72% (truncation at aa 260) | CTX-M-15; SHV-11 | Not detected | Not detected | Not detected | Not detected | [52] |
| SRR10087082 | KP118 | Milan, Italy | 2017 | Not reported | 395 | KL2 | O1ab | OmpK35-72% | TEM-1D; | ybt 16; ICEKp12 | iuc 1 | Not detected | Not detected | Not reported |

| | | | | | | | | | | | | | | |
|---|---|---|---|---|---|---|---|---|---|---|---|---|---|---|
| | | | | | | | | (truncation at aa 260) | KPC-3; SHV-11 | | | | | |
| SRR10087133 | KP469 | Milan, Italy | 2017 | Not reported | 395 | KL2 | O1ab | OmpK35-72% (truncation at aa 260) | TEM-1D; OXA-1; CTX-M-15; KPC-3; SHV-11 | ybt 16; ICEKp12 | iuc 1 | Not detected | Not detected | Not reported |
| ERR11786073 | 08_II | Milan, Italy | 2021 | Urine | 395 | KL2 | O1ab | OmpK35-72% (truncation at aa 260) | TEM-1D; OXA-1; CTX-M-15; KPC-3; SHV-11 | ybt 16; ICEKp12 | iuc 1 | Not detected | Not detected | [53] |

“Not reported” denotes unavailable metadata; “Not detected” denotes an absent determinant in the supplied analysis.

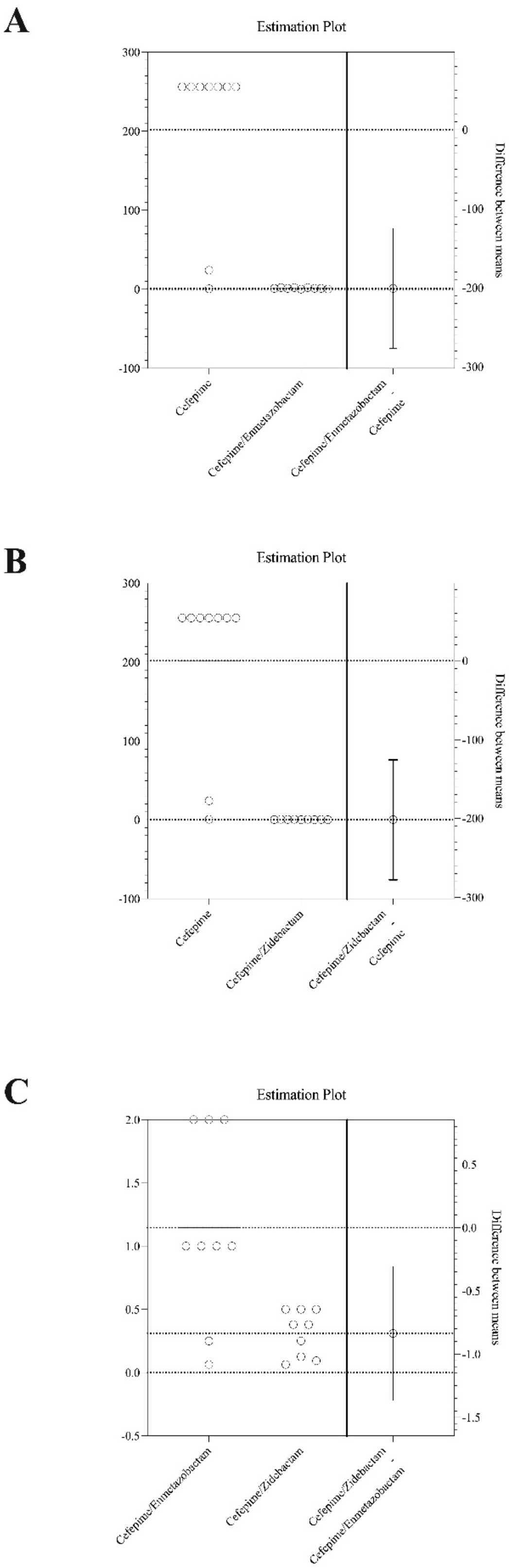


**Figure S1.** Estimation plots of the MICs between cefepime vs cefepime/enmetazobactam (Panel A), cefepime vs cefepime/zidebactam (Panel B), and cefepime/enmetazobactam vs cefepime/zidebactam (Panel C) against OXA-48-producing *Klebsiella pneumoniae* strains included in this study

**Table S2.** Antimicrobial susceptibility profiles of novel antimicrobials and comparator against selected strains.

| Strains | Cefepime (mg/L) | Cefepime/Enmetazobactam (mg/L) | Cefepime/Zidebactam (mg/L) |
|---|---|---|---|
| Kp635 | 256 | 1 | 0.125 |
| Kp448 | 0.5 | 0.25 | 0.064 |
| Kp575 | 256 | 1 | 0.38 |
| Kp325 | 256 | 1 | 0.5 |
| Kp191 | 24 | 0.064 | 0.094 |
| Kp891 | 256 | 2 | 0.25 |
| Kp197 | 256 | 2 | 0.38 |
| Kp15 | 256 | 1 | 0.5 |
| Kp78 | 256 | 2 | 0.5 |

**Table S3.** Assembly statistics of the genomes of the OXA-48-producing *Klebsiella pneumoniae* strains included in this study.

| Sample | Genome completeness | N° contigs | Largest contig (bp) | Total length (bp) | GC (%) | N50 | N90 |
|---|---|---|---|---|---|---|---|
| Kp15 | 99,4 | 82 | 856367 | 5688963 | 57,09 | 342645 | 126663 |
| Kp84 | 99,4 | 56 | 1323887 | 5558493 | 57,14 | 730791 | 104098 |
| Kp137 | 99,4 | 112 | 1035721 | 6063502 | 56,57 | 378621 | 87907 |
| Kp175 | 99,4 | 62 | 1076775 | 5675011 | 57,07 | 827992 | 98024 |
| Kp315 | 99,4 | 86 | 761158 | 5618457 | 57,18 | 281042 | 89420 |
| Kp325 | 99,4 | 91 | 812626 | 5716186 | 57,07 | 368464 | 90465 |
| Kp331 | 99,5 | 96 | 1039827 | 5624672 | 57,11 | 337134 | 89154 |
| Kp383 | 99,4 | 102 | 803664 | 5696659 | 57,07 | 329813 | 77460 |
| Kp573 | 99,4 | 87 | 839216 | 5672164 | 57,08 | 337397 | 68054 |
| Kp635 | 99,4 | 90 | 838569 | 5680456 | 57,11 | 379263 | 89031 |
| Kp797 | 99,4 | 78 | 3019404 | 5910466 | 56,67 | 3019404 | 211558 |
| Kp891 | 99,4 | 50 | 3051735 | 5878073 | 56,7 | 3051735 | 139505 |
| Kp7331 | 99,4 | 68 | 2479891 | 5670779 | 57,11 | 807094 | 380662 |

**Table S4.** Virulence factors among OXA-48-producing *Klebsiella pneumoniae* strains included in this study .

| Isolate | Virulence factors | | | | | | | |
|---|---|---|---|---|---|---|---|---|
| | **Aero-bactin** | **Coli-bactin** | **Enterobactin** | **RmpA DC** | **Rmp A2** | **Salmo-chelin** | **Yer-siniabac-tin** | **Type 1 fim-briae** |
| Kp15 | - | - | *entA, entB, entC, entE, entF, entS, fepA, fepB, fepC, fepD, fepG* | - | - | - | *ybt*16, ICEKp12 | *fimA, fimB, fimC, fimD, fimE, fimG, fimH, fimI* |
| Kp84 | - | - | *entA, entB, entC, entE, entF, entS, fepA, fepB, fepC, fepD, fepG* | - | - | - | *ybt*16, ICEKp12 | *fimA, fimB, fimC, fimD, fimE, fimG, fimH, fimI* |
| Kp137 | - | - | *entA, entB, entC, entE, entF, entS, fepA, fepB, fepC, fepD, fepG* | - | - | - | *ybt*16, ICEKp12 | *fimA, fimB, fimC, fimD, fimE, fimG, fimH, fimI* |
| Kp175 | - | - | *entA, entB, entC, entE, entF, entS, fepA, fepB, fepC, fepD, fepG* | - | - | - | *ybt*16, ICEKp12 | *fimA, fimB, fimC, fimD, fimE, fimG, fimH, fimI* |
| Kp315 | - | - | *entA, entB, entC, entE, entF, entS, fepA, fepB, fepC, fepD, fepG* | - | - | - | *ybt*16, ICEKp12 | *fimA, fimB, fimC, fimD, fimE, fimG, fimH, fimI* |
| Kp325 | - | - | *entA, entB, entC, entE, entF, entS, fepA, fepB, fepC, fepD, fepG* | - | - | - | *ybt*16, ICEKp12 | *fimA, fimB, fimC, fimD, fimE, fimG, fimH, fimI* |
| Kp331 | - | - | *entA, entB, entC, entE, entF, entS, fepA, fepB, fepC, fepD, fepG* | - | - | - | *ybt*16, ICEKp12 | *fimA, fimB, fimC, fimD, fimE, fimG, fimH, fimI* |
| Kp383 | - | - | *entA, entB, entC, entE, entF, entS, fepA, fepB, fepC, fepD, fepG* | - | - | - | *ybt*16, ICEKp12 | *fimA, fimB, fimC, fimD, fimE, fimG, fimH, fimI* |
| Kp573 | - | - | *entA, entB, entC, entE, entF, entS, fepA, fepB, fepC, fepD, fepG* | - | - | - | *ybt*16, ICEKp12 | *fimA, fimB, fimC, fimD, fimE, fimG, fimH, fimI* |
| Kp635 | -- | -- | *entA, entB, entC, entE, entF, entS, fepA, fepB, fepC, fepD, -fepG* | -- | -- | -- | *ybt*16, ICEKp12 | *fimA, fimB, fimC, fimD, fimE, fimG, fimH, fimI* |
| Kp797 | - | - | *-entA, entB, entC, entE, entF, -entS, fepA, fepB, fepC, fepD, fepG* | - | - | - | *ybt*16, ICEKp12 | *fimA, fimB, fimC, fimD, fimE, fimG, fimH, fimI* |

| | | | | | | | | |
|---|---|---|---|---|---|---|---|---|
| Kp891 | - | - | *entA, entB, entC, entE, entF, entS, fepA, fepB, fepC, fepD, fepG* | - | - | - | *ybt*16, ICEKp12 | *fimA, fimB, fimC, fimD, fimE, fimG, fimH, fimI* |
| Kp7331 | - | - | *entA, entB, entC, entE, entF, entS, fepA, fepB, fepC, fepD, fepG* | - | - | - | *ybt*16, ICEKp12 | *fimA, fimB, fimC, fimD, fimE, fimG, fimH, fimI* |

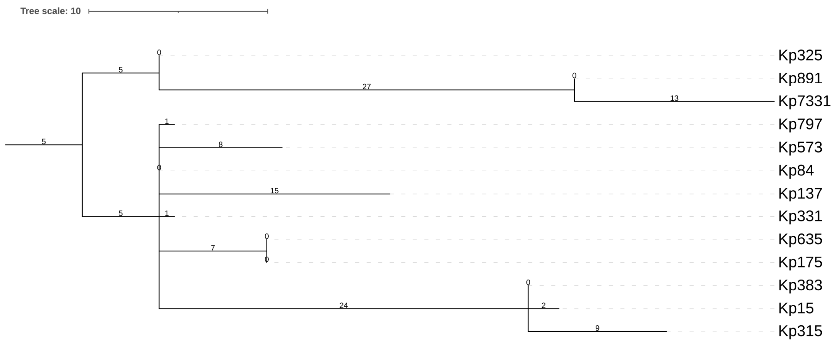


**Figure S2.** Bayesian time-calibrated phylogeny (MCC tree) of the 13 OXA-48-producing *K. pneumoniae* isolates within the broader ST395 reference set. The tree incorporates sampling dates to estimate divergence times; node labels report posterior support and branches are scaled to time. The study cluster and its ancestral nodes are highlighted to summarise the inferred temporal spread in the regional/global context.

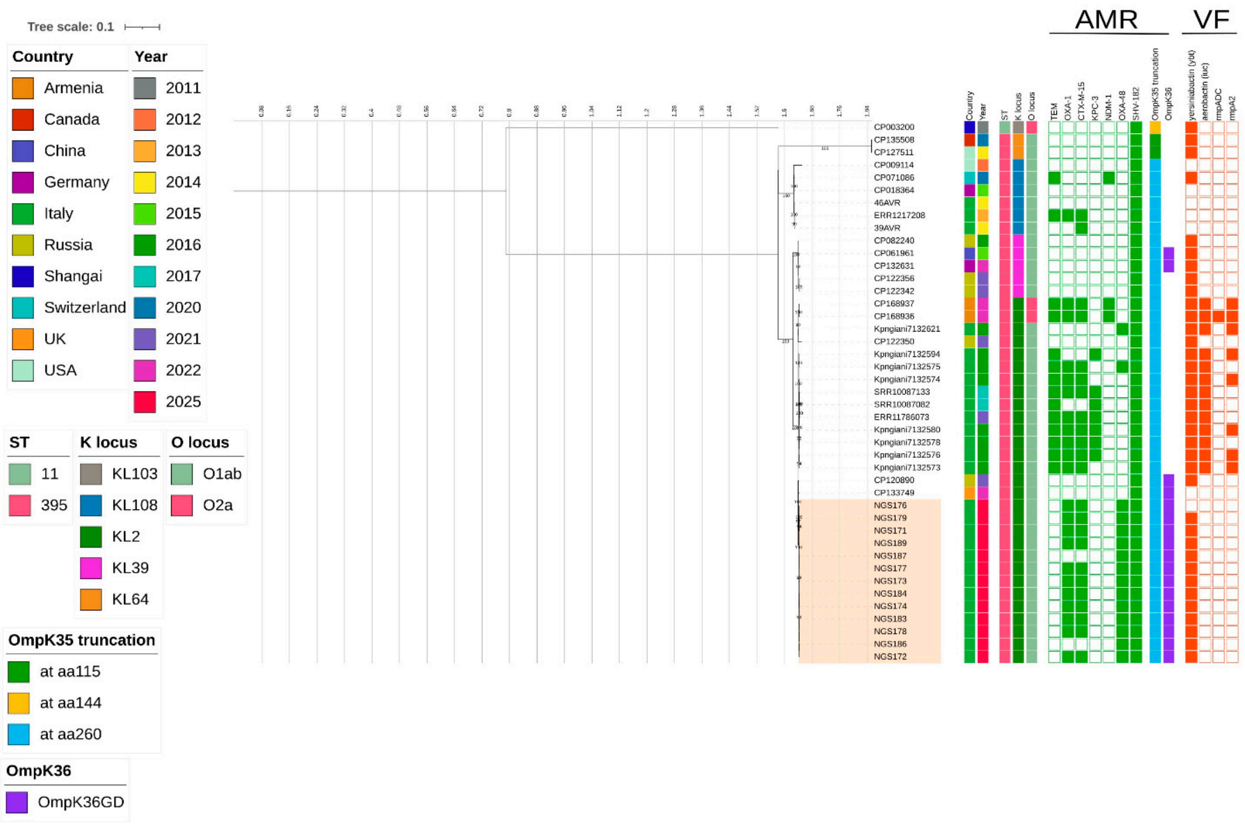


**Figure S3.** Maximum-likelihood phylogeny of the 13 OXA-48-producing *K. pneumoniae* isolates and a set of publicly closest reference genomes available in GenBank. The tree was inferred with IQ-TREE under the K3Pu+F+ASC+R2 model using 1,000 ultrafast bootstrap replicates; bootstrap support values >90 are shown at the corresponding nodes. Tip labels indicate genome identifiers (accession/sample IDs). Annotations depict country and year of isolation, sequence type, K and O loci, presence/absence of antimicrobial resistance and virulence factor genes, and OmpK35 truncation sites and the OmpK36GD porin alteration.
